# Pandemic, Civil Unrest, and a Tropical Storm – What Is Next? The Locusts??

**DOI:** 10.31486/toj.20.5014

**Published:** 2020

**Authors:** Ronald G. Amedee, Margaret Malone

**Affiliations:** ^1^ Designated Institutional Official, Office of Graduate Medical Education, Division of Ochsner Academics, Ochsner Clinic Foundation, New Orleans, LA; Head of Clinical School and Professor, The University of Queensland Faculty of Medicine, Ochsner Clinical School, New Orleans, LA; Editor-in-Chief, *Ochsner Journal*; ^2^ The University of Queensland Faculty of Medicine, Ochsner Clinical School, New Orleans, LA

A lot has happened to each of us and our respective communities since the spring edition of the *Journal* was published. COVID-19 has certainly impacted each of us—from social distancing, to remote working conditions, to wearing masks, to the judicious use of hand sanitizer. Never in the history of modern medicine has teamwork been more important to ensuring optimal patient outcomes. Research focused on the virus has led to new and important revelations about the disease—especially about how to prevent its spread—and for those who test positive, how to treat the disease to prevent the development of dire complications.

This issue of *Ochsner Journal* explores the pandemic from multiple perspectives, with an impressive array of COVID-19–related articles in a special section called Pandemic Perspectives @OchsnerHealth that detail how Ochsner significantly contributed to a concerted Louisiana response that, at the time of this writing, has led to a phase 2 statewide reopening effort. One of those significant contributions was in diagnostic testing. Our cover image shows members of Ochsner's SARS-CoV-2 diagnostics laboratory preparing patient samples for analysis and discussing results. In the top left panel, Medical Laboratory Technician Almedina Tursunović aliquots patient samples to prepare for analysis while fellow Medical Technologist Rianne Ricarte takes notes. The photo in the bottom left panel captures a discussion of test results among Molecular Genetic Pathologist Tong Yang, MD (middle), Clinical Microbiologist Andrea Linscott, PhD (left), and Senior Medical Technologist Luke Caruso (right). In the right panel*,* Luke Caruso sterilizes the laboratory's high throughput RNA extraction instrument before loading patient samples. Our medical students played a huge role in the Ochsner Health response to the pandemic, and you will learn about their contributions in the articles in our special section. My coauthor this month, Margaret “Marge” Malone, is a third-year University of Queensland-Ochsner Clinical School medical student who penned the poem reprinted below as a personal reflection on her participation in the medical response to the crisis.

The senseless and brutal killing of George Floyd has cast a spotlight on opportunities for improvement in racial equality and related healthcare disparities. Each of us must listen to these issues, not only with our ears but also with open minds and hearts.

The first of June signals the beginning of the hurricane season. By the end of the first week of the month, we had already had our C storm as Cristobal swept over the southeast coast of Louisiana.

In positive news, this issue of the *Journal* marks the debut of a new quarterly column on sports medicine. The column is designed to have broad appeal, featuring topics that will be of interest to physicians in an array of specialties.

Finally, in this issue, we pay our respects to the lives and contributions of two medical leaders well known to Ochsner Health. Dr David Margolin (inside front cover) was a valued member of the Ochsner team in colon and rectal surgery, a friend, a colleague, and a gifted clinician-leader. Dr Ethel Weinberg (inside back cover) was the founder of the Alliance for Independent Academic Medical Centers for which the *Ochsner Journal* serves as the publication arm for its members and various proceedings, including the National Initiatives. The deaths of these two individuals have left huge holes in our lives and in those of their families and the many others who knew them best. Their lives were full and meaningful, and the lives of many were enriched by having known and worked with them.

The events of the first half of 2020 have led to an extremely challenging time in our nation's history. We are facing a financial crisis, a health crisis, and a social crisis. Never has there been a more important time for us to discuss how physicians can be part of the solutions needed to help and heal us.

## A COVID-19 Reflection

When I was young and the sky would go dark

Storm sirens wail, ears pound, hair on end

It was easy to know what to do.

Bring in the trash can and anything that floats

Pots and pans under all the old roof leaks

Baby brother and sister down to the basement

Storm radio and flashlights, stories and songs.

Call my parents to see if they had heard.

Maybe storms were wild and vast

But I, little though I was, kept my family safe.

Now we are older and scattered,

Family's safety out of my control –

The storm is a sickness, its onset deadly and silent.

And I don’t know what to do.

I can’t shelter siblings, little, scared, and trusting

Nor can I yet save a single one

Of the hundred thousand strangers dying.

I can quarantine myself – but the power I had

To spread my wings and protect someone

Is lost in the face of this strange storm.

All I can do is soak up what I can

So that when the next storm comes

And I have the strange power of a physician

I can do what I started all this to do

Know what I need to know

And may be able to take, of the hundred thousand,

At least one into the shelter.

–Marge Malone

